# Characterization of complex renal cysts in hereditary leiomyomatosis and renal cell cancerUsing magnetic resonance based qualitative features

**DOI:** 10.1007/s00261-025-05154-w

**Published:** 2025-09-15

**Authors:** Ali Sheikhy, Aryan Zahergivar, Mahshid Golagha, Xiaobai Li, Nikhil Gopal, Fatemeh Homayounieh, Mark W. Ball, Evrim Turkbey, W. Marston Linehan, Ashkan A. Malayeri

**Affiliations:** 1 National Institutes of Health, Bethesda, USA; 2 Internal Medicine Department, George Washington University Hospital, Washington D.C., USA; 3 National Cancer Institute, Bethesda, USA

**Keywords:** HLRCC, Renal cell carcinoma, Renal cysts, MRI

## Abstract

**Purpose:**

Hereditary Leiomyomatosis and Renal Cell Cancer (HLRCC) is a hereditary cancer syndrome associated with germline pathogenic variants of the fumarate hydratase (FH) are at risk for the development of benign renal cysts as well as an aggressive form of renal cell carcinoma which can occur inside the cysts. This study was conducted in order to assess the role of MR imaging characteristics of HLRCC-associated cystic lesions for distinguishing benign from malignant complex renal cysts in this patient population.

**Methods:**

This IRB-approved retrospective study included 42 HLRCC patients (mean age, 46 ± 14 years; men: women, 22:20) with a pathogenic FH germline variant with renal cysts on abdominal MRI. Between June 2002 and May 2022 these patients underwent partial or radical nephrectomy for surgical removal of 76 renal lesions suspicious for renal carcinomas. Two abdominal radiologists independently reviewed the MRI images of all lesions while blinded to the surgical pathology. The lesion characteristics, including location, 3D dimensions, internal composition, characteristics of the cyst wall, nodules, septations, enhancement patterns in different series and restricted diffusion on ADC, and b-2000 series were recorded.

**Results:**

Out of the 76 histologically characterized renal lesions, 44 (58%) were found to be benign and 32 (42%) were malignant. Malignant cystic lesions had a significantly larger mean diameter (4.0 ± 3.4 cm) compared to benign lesions (1.8 ± 2.1 cm, p = 0.002). Inter-reader agreement analysis identified 12 imaging features with moderate agreement (κ >0.4). Univariate analysis identified 8 significant predictors of malignancy: “combined areas of enhancement on T1-weighted images during the nephrogenic phase (the nephrogenic phase, occurring approximately 70 seconds after intravenous contrast injection)”, “endophytic/exophytic mass”, “presence of a nodule”, and “nodule enhancement on T1 nephrogenic phase.” The final multivariable model for Reader 1 achieved an AUC of 0.86 and for reader 2 with an AUC of 0.91, indicating high diagnostic accuracy. At a predicted‐probability threshold of 0.17 (point = 60), the nomogram identified all malignant lesions and would have spared 57% of patients with benign cysts from unnecessary surgery.

**Conclusion:**

Qualitative MRI features, including nodule presence, enhancement patterns, and lesion size, effectively differentiate between benign and malignant renal complex cysts in patients with HLRCC. The final multivariable model achieved high diagnostic, highlighting the potential of MRI in guiding clinical decision-making and improving management of cystic renal lesions in this high-risk population.

**Graphical abstract:**

Created in BioRender. Sheikhy, A. (2025) https://BioRender.com/w45f229

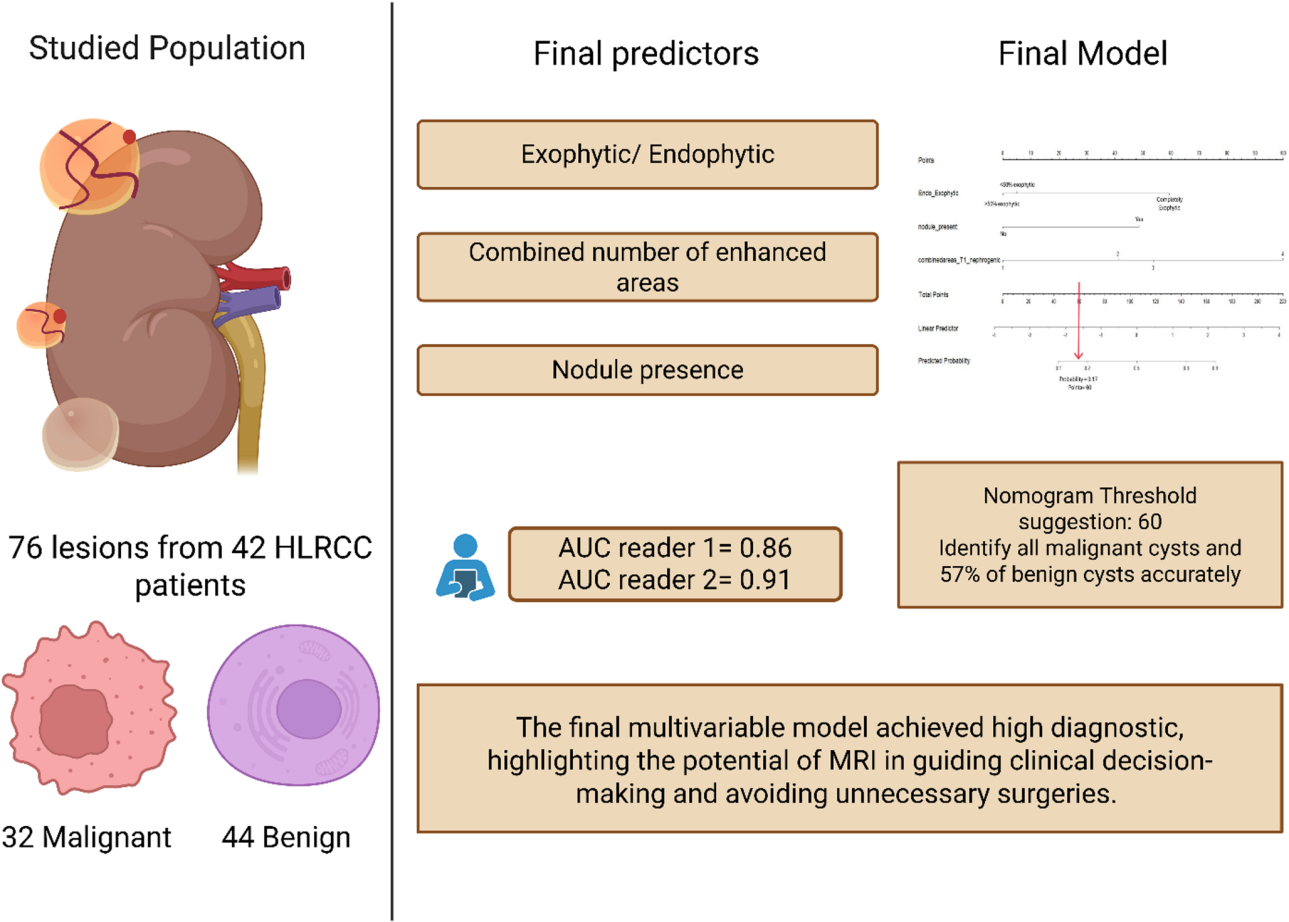

**Supplementary Information:**

The online version contains supplementary material available at 10.1007/s00261-025-05154-w.

## Introduction

Hereditary Leiomyomatosis and Renal Cell Carcinoma (HLRCC), a hereditary cancer syndrome characterized by germline pathogenic alteration in the *fumarate hydratase* (*FH*) gene, in which affected individuals are at risk for the development of benign renal cysts as well as a 15–20% lifetime risk of developing aggressive renal tumors which may be solid or cystic, making it a challenging diagnostic and surveillance problem [1, 2]. One of the primary difficulties in managing patients with HLRCC is distinguishing benign cysts from malignant cystic tumors during screening and surveillance, as the implications for management are significant as HLRCC-associated renal cell carcinoma can develop early and has a propensity to progress rapidly [1, 3–5]. Many of the detected renal cystic lesions are benign. This underscores the critical need for accurate differentiation, as unnecessary interventions can be avoided in patients with benign lesions, while timely treatment of malignant lesions remains crucial.

As HLRCC-associated renal tumors have a propensity to spread when they are small, they require prompt surgical intervention rather than surveillance. Once a suspicious renal lesion is identified, open surgical intervention with wide margins is most often recommended [6, 7].

Annual contrast-enhanced MRI with 1 to 3 mm slice thickness to detect new lesions or changes in previously determined benign lesions under surveillance are recommended for affected individuals [3, 6]. Recent studies have shown that complex renal cysts are highly prevalent, occurring in approximately 50% of family members of HLRCC patients [8]. Unlike other hereditary cancer syndromes, patients with HLRCC often present with high grade unilateral and solitary tumors. Imaging findings regarding HLRCC-associated renal lesions are highly varied, with differences in T2 hyperintensity and contrast enhancement [9]. As a result, predicting the malignant potential of a newly diagnosed renal lesion often proves difficult. Previous studies have highlighted various MRI features that might help differentiate between benign and malignant renal lesions in normal population with incidentally detected renal lesions [10]. These include lesion size, shape, diffusion and enhancement patterns, and internal characteristics such as septations or solid components [9, 11–13]. However, the reliability and predictive value of these features, specifically in patients with HLRCC has not been studied yet.

We performed the current study to systematically assess the utility of qualitative MRI characteristics in distinguishing the pathology of complex renal cysts in HLRCC in order decrease the unnecessary burden of interventions in these patients.

## Methods

### Approvals and disclosures

This retrospective study was conducted under a protocol approved by the Institutional Review Board and was compliant with the Health Insurance Portability and Accountability Act (HIPAA). Written informed consent was obtained from each research participant prior to enrollment in this protocol (NCT00050752, NCT00026884).

### Patient selection

We reviewed our electronic medical record (EPIC, EPIC System Corporation) and identified 71 out of 507 individuals under surveillance with a germline pathogenic variant in the *FH* gene consistent with HLRCC, who had renal cysts between June 2002 and May 2022. We excluded patients who did not undergo partial or radical nephrectomy for histology proof (*n* = 15), did not have contrast-enhanced abdominopelvic MRI prior to the surgery (*n* = 8), or had poor quality MRI (*n* = 6). The final study comprised of 42 patients (mean age, 46 ± 14 years; 24 men, 21 women) with 76 histologically characterized renal lesions (Table 1). We exported clinical information for each patient, including their age, gender, body mass index (BMI), prior renal surgery, and their gene alteration from our electronic medical record.

**Table 1:** Demographic characteristics of studied lesions

		Classification		*P*- value
		Benign	Malignant	
Number of lesions		44	32	**-**
Diameter in cm (mean ± SD)		1.8 ± 2.1	4.0 ± 3.4	0.002
Side (left: right)		24:20	20:12	0.488
Bosniak classification (Reader1 and 2)	I	16	2	< 0.001
		21	1	
	II/IIF	2	0	
		2	0	
	III	23	10	
		9	5	
	IV	3	20	
		12	26	< 0.001

### Image acquisition protocols

All patients underwent standard-of-care and clinically indicated contrast-enhanced abdominal MRI. MRI examinations were performed using systems from three major commercial vendors: GE, Philips, and Siemens, with images acquired both at the NIH and external institutions, including 4 cases on GE (Genesis Signa 1.5 T, Signa Excite 1.5 T, Discovery MR750 3.0 T, Signa HDxt 1.5 T), 13 cases on Philips (Achieva (1.5 T, 3.0 T)), and 25 cases on Siemens (Verio 3.0 T, Symphony 1.5 T, Biograph 3.0 T, Aera 1.5 T, Avanto 1.5 T). Imaging sequences included T2-weighted images, in- and out-of-phase, diffusion-weighted images with corresponding ADC maps (calculated using b-values of 0, 250, and 800 s/mm^2^), and diffusion weighted imaging (DWI) with b-value of 2000 s/mm^2^, and pre-and post-contrast T1-weighted images during corticomedullary phase (20-sec), nephrographic phase (70-sec), and excretory phase (3-min). Seven studies did not have ADC maps, and 15 studies did not have the b-2000 series.

### Qualitative image assessment

Prior to initiating the full study, two abdominal radiologists (AAM and EBT with 14 and 8 years of experience, respectively) conducted a pilot study to optimize the inter-reader reliability. They reviewed MR images of patients who did not have histology proof (15 patients with 19 lesions), discussed discrepancies between the findings, and established agreement on feature definitions.

Then, radiologists independently assessed MR images of all lesions in the full study while they were blinded to the pathology. They recorded location; 3-dimension measurements, 2-dimensions on axial, and 1-dimension on coronal view); internal lesion composition; characteristic features of cyst wall, nodules, and septations (if any) based on the 2019 Bosniak Classification System [14]; degree of lesions enhancement; and percentage of restricted diffusion on ADC map and b-2000 series (Supplementary). The location of the lesions with respect to the sinus lines and polar lines was assessed on the coronal view according to the R.E.N.A.L scoring system [15].

#### Statistical analysis

Mean and standard deviation (SD) were computed to summarize the maximum diameter of the lesions. Other categorical variables were summarized by frequency and percentages. Inter-reader agreement for the imaging parameters was assessed using the kappa statistic. Due to the clustering nature of the data where multiple lesions were taken from the same subjects, a repeated measures approach was employed to account for the correlated observations [16]. Variables with the lower bound of the 95% confidence interval (CI) for the kappa statistic greater than 0.4, which indicates moderate agreement [17], were considered to exhibit at least moderate agreement between raters. estimating equations (GEE) with an exchangeable working-correlation matrix and robust “sandwich” standard errors, clustering on medical-record number (MRN). The statistical significance of each feature in these univariate GEE models was assessed with the Wald χ² test; predictors with a Wald *P* < 0.10 were retained for multivariable evaluation. Multivariable model building then proceeded with forward stepwise addition: at every step the candidate variable that produced the greatest decrease in the quasi-likelihood information criterion corrected for small samples (QICu) was incorporated, and the process stopped once no remaining predictor further lowered QICu. The final model therefore minimized QICu while retaining only variables whose Wald P remained below 0.10, balancing parsimony with clinical relevance. A nomogram was subsequently constructed based on the final multivariable model to provide individualized risk estimates for positive pathology outcomes. This nomogram translates the regression coefficients into an easy-to-use graphical tool, enabling clinicians to calculate the probability of a positive result by assigning weighted scores to each predictor. All statistical analyses were performed using SAS (SAS Institute Inc., Cary, NC, USA, version 9.4) and R (version 4.3.2) with the “**geepack**” package [18].

## Results

### Study characteristic

**Fig. 1:**
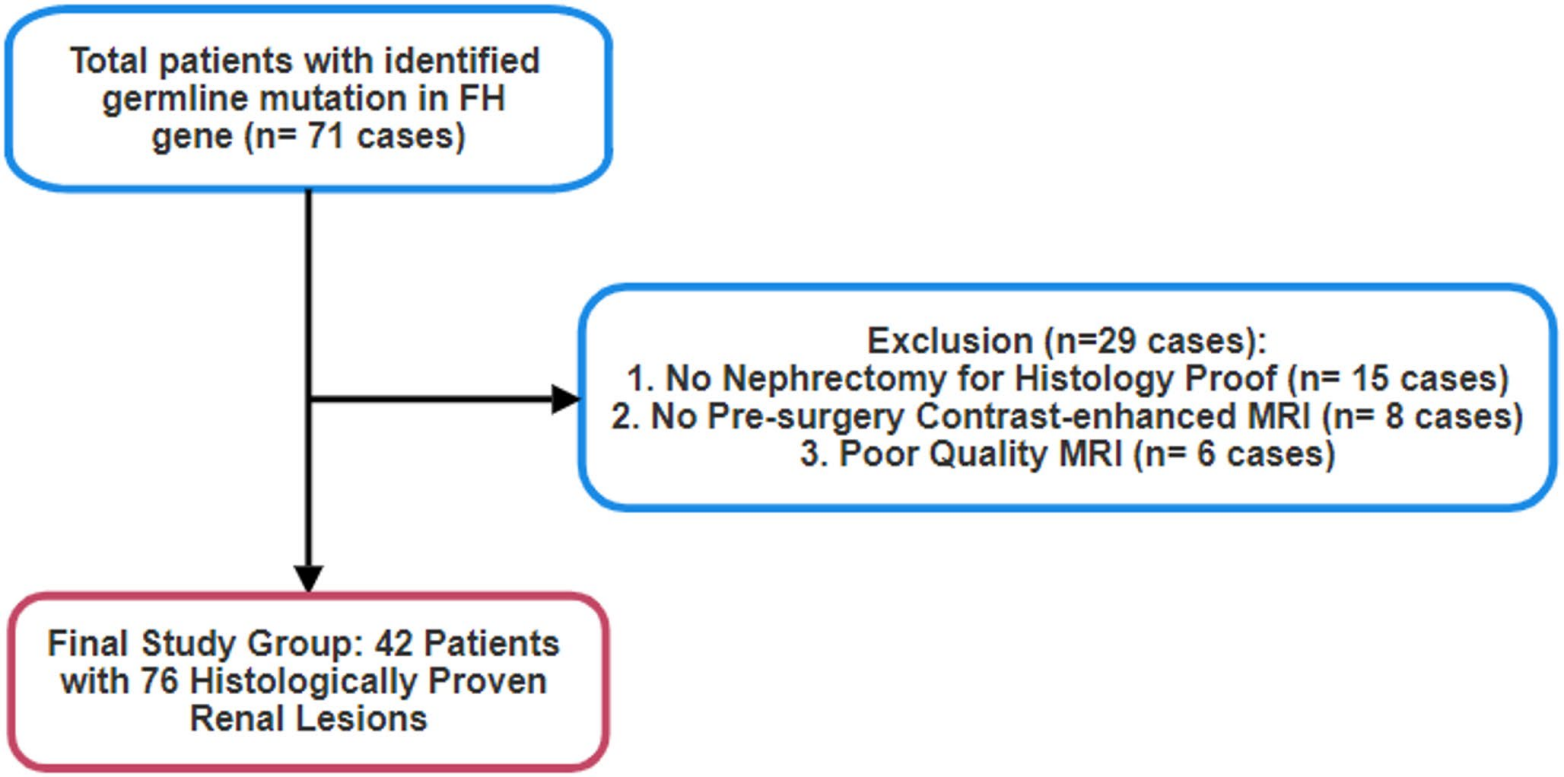
Study flowchart

Of the 76 renal lesions evaluated (Fig. 1), 44 (58%) were benign, while 32 (42%) were malignant. Malignant lesions had significantly larger dimensions (mean ± SD: 4.0 ± 3.4 cm) compared to benign lesions (1.8 ± 2.1 cm, *p* = 0.002; Table 1). A total of 18 patients (43%) had only benign cystic lesions, 7 patients (17%) had both malignant and benign lesions, and 17 patients (40%) had exclusively malignant renal lesions (Table 2).

**Table 2:** Demographic characteristics of studied individuals

	Classification			*P*-value
	Benign (*n* = 18)	Mixed (*n* = 7)	Malignant (*n* = 17)	
Age (mean ± SD)	46 ± 17	44 ± 13	48 ± 15	0.840
Gender (M: F)	9:9	4:3	9:8	0.950
BMI (mean ± SD)	29.5 ± 8.2	30.5 ± 10.1	32.2 ± 8.9	0.624

### Inter-reader agreement analysis

**Fig. 2:**
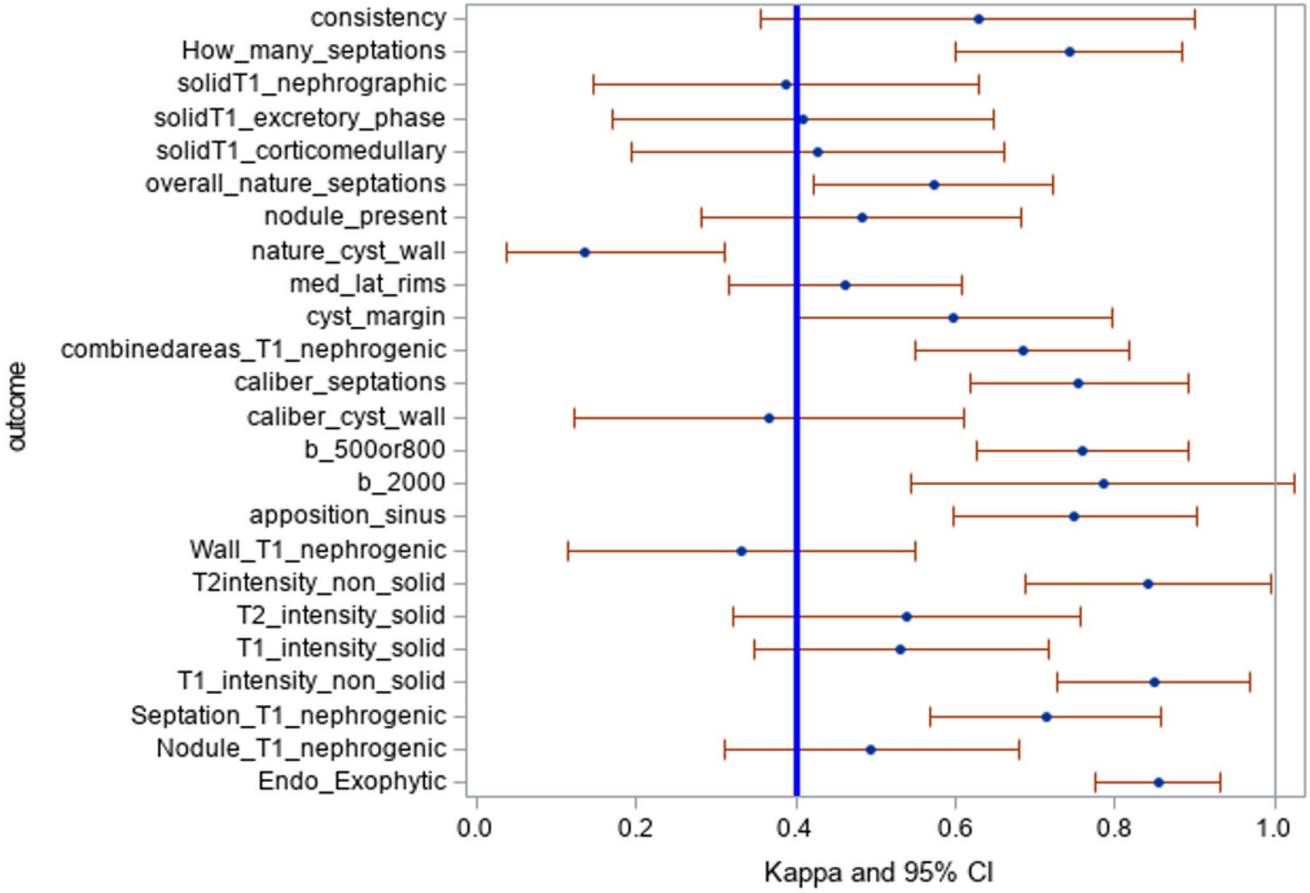
Clustered Kappa value of studied variables

Inter-reader reliability was assessed using kappa statistics, with 12 imaging features demonstrating moderate agreement (κ > 0.4, 95% CI). These included “number of septations,” “combined areas of enhancement on T1 nephrogenic phase,” “diffusion restriction of the mass on ADC maps,” and “nodule presence” (Supplementary Table 1). These variables were entered into the univariate analysis (Fig. 2).

### Univariate analysis

Raw counts and percentages of malignant and benign lesions for each predictive radiologic feature reported on Table S2. All 12 variables from the previous step were included in the univariable analysis model. Data from each reader were analyzed separately. The results indicated that nine variables, ‘maximum diameter”, “Exophytic/Endophytic lesion”, “number of septations”, “septation nature”, “combined areas of enhancement on T1 nephrogenic phase”, “septation enhancement on T1 nephrogenic phase”, “ Diffusion restriction of the mass on ADC maps”, “nodule presence”, and “nodule enhancement on T1 nephrogenic phase”, showed a p-value less than 0.1 for both readers (Table 3). For the first reader, ADC values exceeding those of normal renal parenchyma were associated with the highest odds ratio for malignancy (OR = 80.67, 95% CI: 7.49–868.01). Moreover, for the second reader, nodule enhancement on the T1 nephrogenic phase was the strongest predictor of malignancy (OR = 35.00, 95% CI: 7.14–171.39).

**Table 3:** Significant variables in univariate model

			Reader 1			Reader 2		
Variable			OR	95% CI	P-value	OR	95% CI	P-value
Maximum diameter			1.37	(1.03, 1.83)	0.031	1.36	(1.02, 1.80)	0.034
Endophytic (Completely endophytic as reference)	Model*		–	–	0.044	–	–	0.003
	< 50% exophytic		2.04	(0.95, 4.34)	0.065	4.34	(12.5, 1.69)	0.005
	> 50% exophytic		5.26	(1.22, 25)	0.026	5.55	(1.69, 16.7)	0.004
Number of septations (Absent as reference)	Model		–	–	0.011	–	–	0.012
	Few (1–3)		2.16	(0.82, 5.67)	0.119	1.34	(0.44, 4.09)	0.603
	Many (> 4)		5.68	(1.79–18.10_	0.003	14.7	(2.24, 96.5)	0.005
Septa’s caliber (no septation as reference)	Model		–	–	0.002	–	–	0.060
	Thin (< 2 mm in thickness)		2.89	(1.08, 7.67)	0.034	1.74	(0.59, 5.07)	0.310
	Minimally thickened (3 mm in thickness)		0.83	(0.17, 4.06)	0.820	2.24	(0.40, 12.5)	0.356
	Thickened (> 4 mm in thickness)		21.30	(3.95, 115)	< 0.001	10.5	(1.93, 57.5)	0.006
Septation nature (absence of septation as the reference)	Model		–	–	0.005	*–*	–	0.108
	Regular		1.89	(0.76, 4.68)	0.172	1.74	(0.53, 5.70)	0.361
	Irregular		8.83	(2.35, 33.20)	0.001	4.61	(1.11, 19.1)	0.035
Combined areas of enhancement T1 nephrogenic (No Enhancement as reference)	Model		–	–	< 0.001	–	–	0.002
	One part enhancement		5.91	(1.21, 29.0)	0.028	3.61	(0.62, 20.8)	0.150
	two parts enhancement		8.89	(1.90, 41.6)	0.005	31.9	(3.73, 273)	0.002
	three parts enhancement		47.1	(8.16, 272)	< 0.001	115	(9.25, 1420)	< 0.001
ADC maps based on b500 or 800 (No diffusion restriction as the reference)	Model		–	–	< 0.001	–	–	0.002
	Less than renal		11.4	(1.39, 93.3)	0.023	4.22	(0.65, 27.48)	0.132
	Same as renal		18.3	(2.02, 166)	0.010	9.49	(1.69, 53.32)	0.011
	More than renal		75.6	(9.80, 583)	< 0.001	23.75	(3.69, 152.87)	< 0.001
Nodule presence			11.90	(3.40, 41.7)	< 0.001	21.4	(5.70 80.1)	< 0.001
Nodule enhancement on T1 Nephrogenic phase			12.1	(3.85, 38.1)	< 0.001	35.20	(8.17, 151)	< 0.001
Wall enhancement on T1 Nephrogenic phase			7.36	(2.66, 20.4)	0.001	5.82	(1.85, 18.3)	0.003
Septation enhancement on T1 Nephrogenic phase			3.23	(1.35, 7.74)	0.008	3.37	(1.15, 9.87)	0.026

*The ‘model’ p-value is the p-value of the overall effect of the variable.

### Multivariable and ROC analysis

**Fig. 3:**
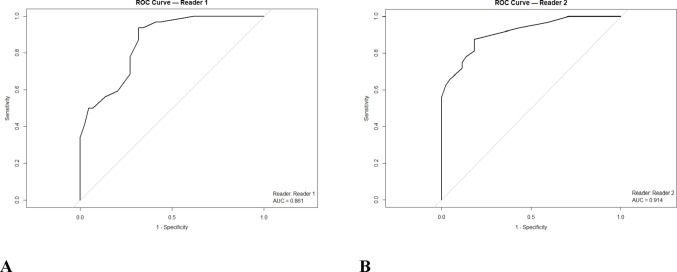
ROC curve for final models for reader 1 (**A**) and 2 (**B**)

**Fig. 4:**
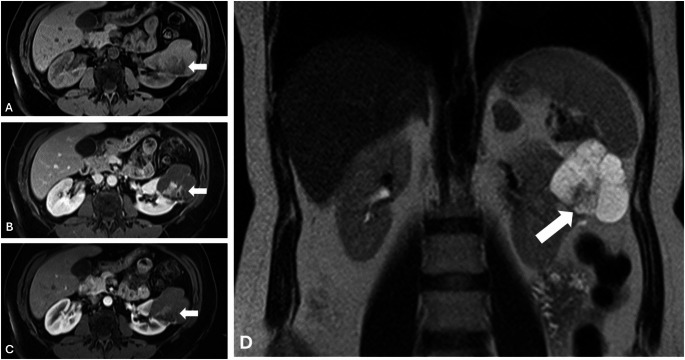
Complex renal cyst in a 54-year-old female with HLRCC. Contrast-enhanced MRI reveals a complex cyst measuring 5.8 cm, featuring a solid component (arrow) visible in the pre-contrast phase (**A**) and on coronal T2 images (**D**), with heterogeneous enhancement noted in the post-contrast nephrogenic (**B**) and delayed (**C**) phases. Final pathology report confirmed the cyst as malignant.

**Fig. 5:**
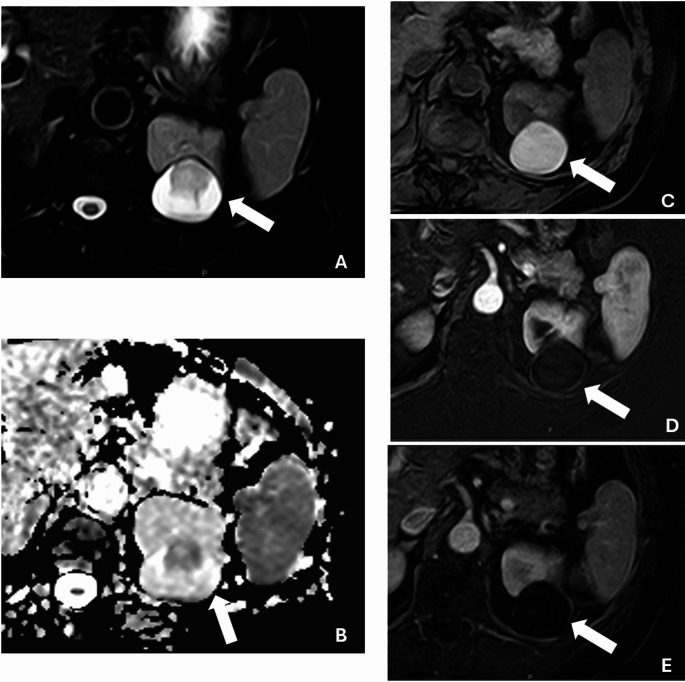
Complex renal cyst in a 68-year-old female with HLRCC. Contrast-enhanced MRI reveals a well-circumscribed complex cyst measuring 4.0 cm (arrows), demonstrating heterogeneous T2 intensity on the T2-weighted image (**A**). The cyst exhibits restricted diffusion on the ADC map (**B**) and appears hyperintense on the pre-contrast phase (**C**). No enhancement is observed on the subtracted images of the post-contrast nephrogenic (**D**) and delayed (**E**) phases. Final pathology confirmed the cyst as benign.

In multivariable logistic regression analysis, statistically significant predictors were identified for both readers. For the first reader, the final model included endophytic lesion (with < 50% exophytic lesion showing an OR of 0.12 [95% CI: 0.02–0.67] and completely endophytic lesion an OR of 0.10 [95% CI: 0.01–0.65]), nodule presence (OR = 7.12 [95% CI: 1.49–33.92]), and combined areas of enhancement on T1 nephrogenic phase (OR = 2.58 [95% CI: 1.21–5.50]); this model achieved an AUC of 0.86 (95% CI: 0.82–0.91; Figs. 3 and 4), meanwhile Bosniak classification performance was 0.79 in this case. For the second reader, the final model comprised endophytic lesion (with < 50% exophytic lesion yielding an OR of 0.07 [95% CI: 0.01–0.49] and completely endophytic lesion an OR of 0.06 [95% CI: 0.01–0.43]), combined areas of enhancement on T1 nephrogenic phase (OR = 4.35 [95% CI: 1.54–12.23]), and nodule enhancement on T1 nephrogenic phase (OR = 6.42 [95% CI: 0.81–50.68]) (Fig. 5; Table 4), with this model demonstrating an AUC of 0.91 (95% CI: 0.85–0.98; Fig. 3), outperforming the Bosniak scoring system, which demonstrated an AUC of 0.81.

**Fig. 6:**
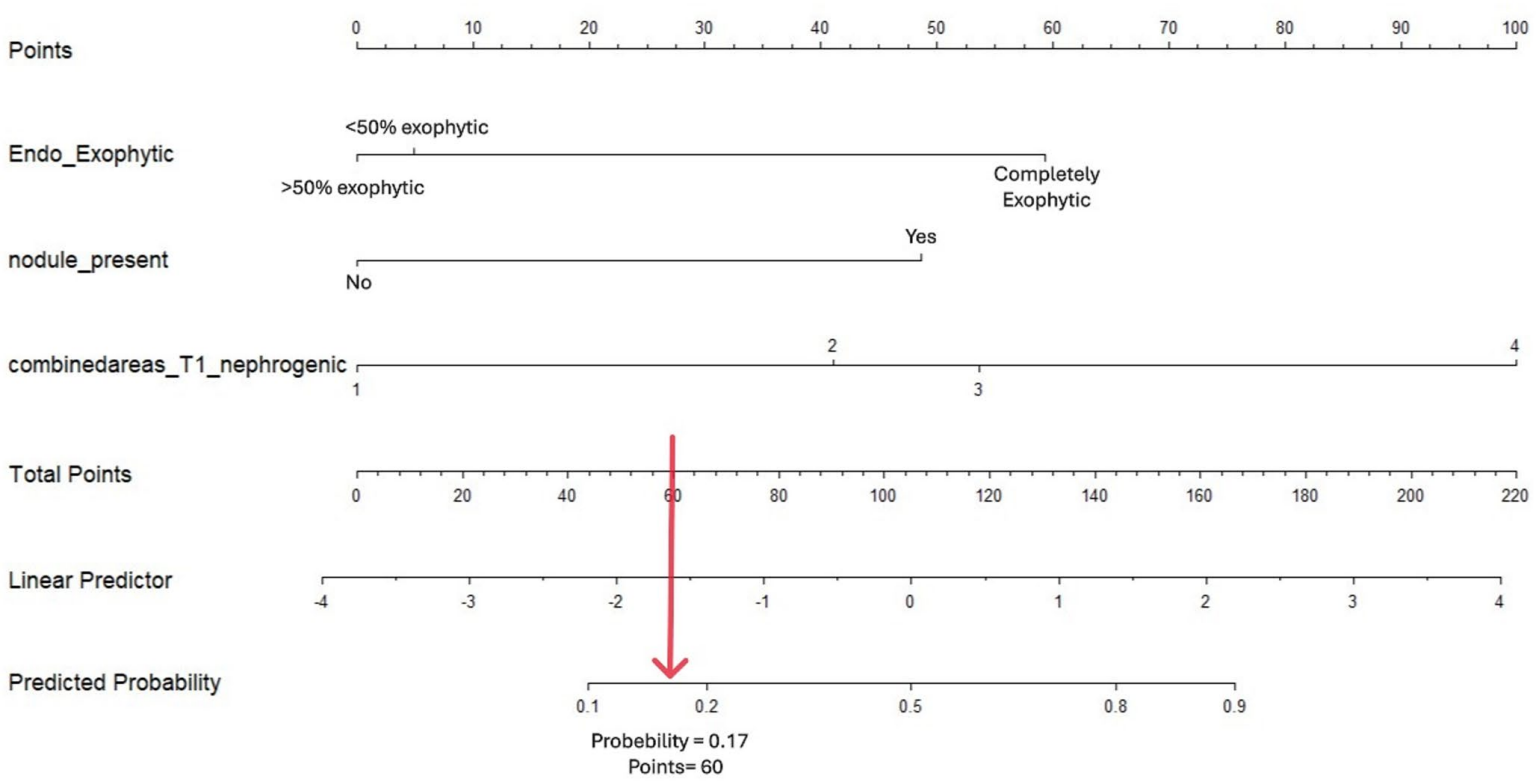
Developed nomogram for qualitative features.

**Table 4:** Final multivariable model (stepwise regression)

		Reader 1			Reader 2		
Variable		OR	95% CI	P-value	OR	95% CI	P-value
Endophytic (Completely endophytic as reference)	< 50% exophytic	6.25	(1.66, 20)	0.005	9.09	(2.12, 33)	0.003
	> 50% exophytic	7.69	(1.21, 4.76)	0.032	8.33	(2.27, 36)	0.001
Combined areas T1 nephrogenic (Absent as the reference)	One part enhancement	12.70	(2.12, 76.3)	0.005	9.52	(1.44, 62.80)	0.019
	Two parts enhancement	9.03	(1.51, 54.00)	0.016	53.00	(4.57, 614)	0.001
	Three parts enhancement	56.40	(9.86, 323.00)	< 0.001	56.80	(4.10, 788)	0.002
Nodule presence		6.13	(1.75, 21.50)	0.004	6.61	(1.12, 39.00)	0.037

### Development and performance of nomogram

To provide clinicians with an easy-to-use tool, we developed a nomogram. Our nomogram performed well, with a suggested probability threshold of 0.17, corresponding to a score of 60 on the nomogram. This threshold effectively identified all malignant cysts while correctly classifying 25 out of 44 benign cysts (57%). This balance between sensitivity and specificity enhances the clinical utility of the model in distinguishing malignant from benign cystic lesions (Fig. 6). 

## Discussion

In this study we found nodule presence or enhancement, exophytic mass, septation enhancement, and total number of enhanced areas as potential predictors of malignancy in cystic lesions of patients with HLRCC. Moreover, univariate analysis revealed that diffusion restriction in the lesion’s solid component exhibited the strongest association with malignancy.

Considering the recommended life-long annual surveillance for patients with HLRCC [19], this study presents significant implications for the clinical management of renal lesions in this population.

Studies have shown that approximately 50% of individuals with HLRCC, including diagnosed patients and their first-degree relatives, develop renal cysts. Notably, only half of these cysts are found to be malignant renal cell carcinomas (RCCs) [8]. HLRCC associated renal lesions are distinct from other types of renal cell carcinoma due to their frequent cystic manifestations, which complicate the differentiation between benign and malignant lesions. This unique presentation underscores the importance of our findings, which highlight the use of MRI findings to improve the characterization and management of these lesions [9, 19].

### Key MRI features in differentiating renal lesions

This study focused on the MRI characteristics of the cystic lesions, and we included the effect of the size in the analysis. While we cannot deny the impact of other features in lesion characterization, our findings highlight the following MRI characteristics that were more prominent in differentiating benign vs. malignant renal lesions in HLRCC.

#### Enhancement and nodule presence in contrast-enhanced nephrogenic phase

The presence of nodule and its enhancement in nephrogenic phase emerged as a significant predictor, aiding in distinguishing between benign and malignant lesions. Proposed Bosniak classification for MRI also indicated that enhancing nodules categorize the cyst as the type IV class, which is highly suspicious for malignancy, with a 90% likelihood of being malignant [14, 20]. HLRCC is primarily caused by a germline mutation in the FH gene. In kidney cancer cells lacking functional FH, fumarate accumulates and inhibits prolyl hydroxylases, leading to the stabilization of hypoxia-inducible factor (HIF). This stabilization upregulates HIF target genes—such as GLUT1, vascular endothelial growth factor (VEGF), platelet-derived growth factor (PDGF), and transforming growth factor (TGF)α—which collectively promote tumor growth [21]. Additionally, malignant tumors often drive angiogenesis via (VEGF) resulting in disorganized vasculature that appears as nodular enhancement on imaging [22, 23]. Said et al. revealed that nodular enhancement on the nephrogenic phase is associated with malignancy in solid renal neoplasm with an AUC of 0.71 [24].

#### Diffusion restriction

While there is no clear consensus on the optimal b-value for evaluating renal lesions, a range of 50 to 1000 is generally recommended [25]. A meta-analysis conducted by Lassel et al. revealed that ADC value is significantly lower in malignant lesions [26]. Several studies have shown the role of higher b-value DWI in detecting malignancy including breast cancer [27], liver metastasis [28], prostate cancer [29], renal cell carcinoma [30], and rectal adenocarcinomas [31]. Previous studies conducted by Chaurasia et al. revealed that higher b-value DW-MRI has high diagnostic accuracy in classifying of renal masses in HLRCC patients [11]. According to their findings the best AUC for the b-800 and b-2000 were 0.756 and 0.992, respectively in diagnosis of malignant renal lesions. Our study demonstrated that ADC maps were better predictors than b2000 images. This difference likely arises from the patient populations: our study focused exclusively on cystic lesions in patients with HLRCC, whereas Chaurasia et al. included both cystic and solid renal masses. Another study by Nikolovski et al. on fumarate hydratase-deficient renal cell carcinoma found that every patient with a diagnosis of RCC exhibited diffusion restriction in the solid component of their tumor [32].

#### Exophytic/endophytic

Our findings suggest that exophytic renal lesions in HLRCC exhibit a higher likelihood of malignancy, potentially due to their distinct tumor microenvironment. While this observation is novel in the context of HLRCC, prior studies have shown that perinephric adipose tissue is an active endocrine organ that secretes pro-angiogenic and pro-inflammatory factors such as IL-6, TNF-α, and VEGF, which can support tumor growth and neovascularization [33, 34]. Additionally, the anatomical proximity of exophytic lesions to capsular vasculature may facilitate earlier vascular recruitment, although this mechanism remains speculative in the setting of HLRCC and has not been directly validated. Importantly, while tumor development in HLRCC is fundamentally driven by FH mutation, it is plausible that lesion location may modulate the local tumor milieu, contributing to more aggressive behavior [35]. A review of the current literature reveals a notable gap regarding dedicated radiologic evaluation of exophytic versus endophytic features specifically in HLRCC. Most published studies and imaging guidelines, EAU guidelines on renal cell carcinoma [28] describe tumor morphology in hereditary renal cancers as part of overall assessments but do not directly compare the prognostic implications of exophytic versus endophytic growth in HLRCC. This lack of focused investigation underscores the need for future prospective studies to determine whether exophytic growth independently predicts a more aggressive behavior in these tumors, which could ultimately refine imaging-based risk stratification and influence clinical management. These findings emphasize the importance of exophytic/endophytic tumor assessment in risk stratification, underscore the potential clinical utility of incorporating growth pattern analysis into surveillance protocols for HLRCC patients. Such an approach may improve early detection of malignant transformation and thereby enhance clinical outcomes in this high-risk population.

#### Septation enhancement

According to Bosniak classification, cysts with one or more enhancing septa are considered intermediate probability of being malignant or class III [14]. According to recent studies, the prevalence of malignancy among Bosniak class III cysts is about 60% [36]. The study by Tse et al. demonstrated that septal or wall enhancement in complex renal cysts has a high sensitivity of 84–87% for predicting malignancy. This suggests that these imaging features are reliable indicators of malignancy in complex cysts [10]. In a study by Gomma et al., septal enhancement in cystic renal lesions was identified as a significant predictor of malignancy [37]. While our findings did not directly replicate this association, we observed that the total number of enhanced areas was significantly linked to malignancy. This suggests that, rather than septal enhancement alone, the overall enhancement within the mass may also indicate malignancy, potentially reflecting a broader tumor vascularization pattern. The findings of this study have significant implications for the diagnosis and management of renal lesions in patients with HLRCC. By identifying MRI features such as nodule enhancement, diffusion restriction, and solid components as potential predictors of malignancy.

Although the 2019 Bosniak classification system provides a widely accepted framework for characterizing complex renal cysts, it was developed for the general population and may not fully capture the unique imaging features observed in hereditary cancer syndromes such as HLRCC. In our study, several qualitative features beyond the standard Bosniak criteria, such as the combined extent of enhancement across multiple regions of the cyst, the lesion’s exophytic or endophytic orientation, and diffusion restriction, were found to be significant predictors of malignancy. These parameters are not explicitly incorporated into the Bosniak system but may offer added diagnostic value in genetically predisposed populations. Our findings suggest that supplemental imaging features tailored to the biologic behavior of HLRCC-associated lesions may enhance risk stratification and clinical decision-making beyond the current classification framework. Our model demonstrated superior diagnostic performance compared to the Bosniak classification system, with almost 10–15% better performance. This suggests that the integration of individual imaging features, such as enhancement patterns and lesion morphology, may enhance diagnostic accuracy beyond conventional Bosniak categorization. Future prospective studies are warranted to validate these findings and explore whether a modified or extended classification system may better serve high-risk hereditary cohorts.

The implementation of this nomogram can significantly influence clinical decisions by minimizing overtreatment. In patients with HLRCC, any indeterminate renal cyst often raises concern due to the syndrome’s aggressive cancer risk – historically leading to surgeries even for lesions that prove benign on pathology [9]​. By accurately identifying malignant and likely benign cysts, the nomogram helps avoid unnecessary surgeries. Qualitative imaging criteria can predict a simple benign cyst​, allowing safe active surveillance instead of immediate excision. Avoiding unnecessary partial nephrectomies or nephron-sparing surgeries spares patients from surgical risks and preserves renal parenchyma. This is crucial because overtreatment and unnecessary removal of benign lesions can cause renal impairment [38, 39]​. The nomogram-guided approach ensures that surgical intervention is reserved for lesions with a high probability of malignancy, thereby reducing the rate of benign cysts being unnecessarily resected. Moreover, the tool enables a more nuanced, patient-specific management plan, intervening when needed and observing when safe​, rather than a “one-size-fits-all” aggressive approach for all cystic lesions.

Our results provide a foundation for future research to refine imaging-based diagnostic criteria and to develop standardized protocols tailored specifically for HLRCC-associated renal lesions. Such advancements could enhance surveillance strategies, optimize patient outcomes, and reduce the burden of invasive procedures.

### Limitations

This study has several limitations. First, its retrospective nature and relatively small sample size may limit the generalizability of the findings. Additionally, variations in MRI techniques and equipment over the study period could introduce inconsistency in imaging results. Although moderate inter-reader agreement was observed for several features, discrepancies in the selection and significance of certain variables between readers were noted. These differences may reflect subjective interpretation of qualitative features, such as enhancement patterns or septal morphology, despite efforts to standardize definitions through a pilot calibration session. Furthermore, heterogeneity in MRI protocols: spanning multiple vendors, field strengths, and institutions, may have affected reader confidence and feature detection. These findings underscore the need for future prospective studies with larger cohorts, harmonized imaging protocols, and the incorporation of quantitative imaging biomarkers to reduce inter-observer variability and enhance reproducibility. Additionally, the relatively small sample size may increase the risk of overfitting in the multivariable model, particularly given the high odds ratios observed; thus, external validation in larger cohorts is warranted.

## Conclusion

This study highlights the effectiveness of qualitative MRI features in distinguishing benign from malignant complex renal cysts in patients with HLRCC. Key imaging characteristics, such as nodule presence, enhancement patterns, and exophytic mass, demonstrated strong predictive value for malignancy. Our multivariable model achieved high diagnostic accuracy, supporting the role of qualitative imaging in early detection and risk stratification. Notably, the use of these features reduced unnecessary surgeries by 43%, optimizing patient management by preventing overtreatment while ensuring timely intervention for malignant lesions. These findings underscore the potential of qualitative MRI assessment to enhance personalized treatment strategies and improve clinical outcomes in HLRCC patients.

## Supplementary Information

Below is the link to the electronic supplementary material.

Supplementary Material 1

## Data Availability

The datasets generated and analyzed during the current study were produced within the National Institutes of Health (NIH) Clinical Center and contain sensitive, individual-level information. In accordance with NIH institutional policies, U.S. federal regulations, and patient-privacy requirements, these data are not publicly available.

## Declaration

Graphical Abstract: Created in BioRender. Sheikhy, A. (2025) https://BioRender.com/w45f229.
