# Supplementary material for: Characterization of complex renal cysts in hereditary leiomyomatosis and renal cell cancerUsing magnetic resonance based qualitative features
Source: Abdom Radiol (NY). 2025 Sep 15;51(3):1459–71. doi: 10.1007/s00261-025-05154-w (PMC12971841; doi:10.1007/s00261-025-05154-w)
Supplement: Supplementary file 1 — Supplementary Material 1 [file 261_2025_5154_MOESM1_ESM.docx]

1. **How well-defined is the tumor margin?**

**1-** Completely well-defined  **2-** Approximately 3/4th of the tumor has a clearly defined margin **3-** Approximately 1/2 of the tumor has a clearly defined margin  **4-** Approximately 1/4 of the tumor has a clearly defined margin **5-** No clearly defined tumor margin can be identifier

1. **>50% exophytic, <50% exophytic, or entirely endophytic?**
2. **Does the tumor involve the renal sinus**? Yes No
3. **Where is the tumor localized with respect to the medial and lateral rims of the kidney?**

**1-** On the lateral rim of the kidney **2-** Near the lateral rim of the kidney **3-** Indeterminate

**4-** Near the medial rim of the kidney **5-** On the medial rim of the kidney

1. **Where is the tumor located with respect to the sinus and polar lines?**

**1-** Entirely above or below the sinus lines **2-** <50% tumor crosses either sinus line **3-** >=50% tumor crosses either sinus line OR is entirely between sinus lines but is entirely above or below polar lines **4 -** <50% of tumor crosses polar line **5 -** >=50% tumor crosses polar line OR lies entirely between polar lines OR tumor crosses axial renal midline

1. **How is the T2 signal intensity of the tumor best characterized?**

1-Same as paraspinal muscles 2- More than paraspinal muscles but less than renal cortex 3- Same as renal cortex 4- More than renal cortex but less than CSF 5- Same as CSF

1. **How is the internal composition of the tumor best described?**

1- Predominantly solid 2-Mixed solid (>50% solid) 3. Mixed cystic (>50% cystic) 4. Predominantly cystic

1. **On nephrogenic phase of post-contrast enhanced series, what is the percent enhancement of the mass (relative to the unenhanced phase)?**

1-0-25% 2-25-50% 3-50-75% 4-75-100%

1. **How does the solid portion of the mass enhance compare to the surrounding renal cortex in each of the enhancement phases?**
   1. Corticomedullary - Less than renal cortex Same as renal cortex More than renal cortex
   2. Nephrographic – Less than renal cortex Same as renal cortex More than renal cortex
   3. Excretory – Less than renal cortex Same as renal cortex More than renal cortex
2. **How is the degree of diffusion restriction of the mass on ADC (based on b-500 or b-800)?** (1=No restriction- same as cerebrospinal fluid (CSF), 2=more than CSF but less than renal cortex, 3=Same as renal cortex, 4=more than renal cortex but less than spleen, 5=Same as spleen)

1 2 3 4 5

1. **How many combined areas of enhancement? (Wall enhancement + Septation enhancement + Nodule enhancement) one point for each one.**

| kappa | lower95 | upper95 | variable |
| --- | --- | --- | --- |
| 0.853244 | 0.774198 | 0.932291 | Endo_Exophytic |
| 0.494398 | 0.3104 | 0.678395 | Nodule_T1_nephrogenic |
| 0.712912 | 0.568919 | 0.856906 | Septation_T1_nephrogenic |
| 0.847878 | 0.726782 | 0.968975 | T1_intensity_non_solid |
| 0.531665 | 0.347252 | 0.716078 | T1_intensity_solid |
| 0.539345 | 0.322581 | 0.756109 | T2_intensity_solid |
| 0.841463 | 0.686777 | 0.99615 | T2intensity_non_solid |
| 0.332846 | 0.114922 | 0.550769 | Wall_T1_nephrogenic |
| 0.749634 | 0.596402 | 0.902867 | apposition_sinus |
| 0.784737 | 0.545437 | 1.024038 | b_2000 |
| 0.760059 | 0.627567 | 0.892551 | b_500or800 |
| 0.367583 | 0.124202 | 0.610964 | caliber_cyst_wall |
| 0.754964 | 0.61761 | 0.892318 | caliber_septations |
| 0.68406 | 0.550379 | 0.81774 | combinedareas_T1_nephrogenic |
| 0.597116 | 0.399217 | 0.795015 | cyst_margin |
| 0.462061 | 0.315363 | 0.608759 | med_lat_rims |
| 0.136364 | 0.037302 | 0.310029 | nature_cyst_wall |
| 0.483169 | 0.2829 | 0.683438 | nodule_present |
| 0.572233 | 0.421861 | 0.722604 | overall_nature_septations |
| 0.427895 | 0.194267 | 0.661524 | solidT1_corticomedullary |
| 0.409871 | 0.171258 | 0.648483 | solidT1_excretory_phase |
| 0.387864 | 0.146609 | 0.62912 | solidT1_nephrographic |
| 0.742736 | 0.600832 | 0.884641 | How_many_septations |
| 0.627922 | 0.355141 | 0.900703 | consistency |

Table S1: Calculated clustered Kappa values

|  | | **Reader 1** | | | **Reader 2** | | |
| --- | --- | --- | --- | --- | --- | --- | --- |
| **Variable** | **Level** | **Benign** | **Malignant** | **P-value** | **Benign** | **Malignant** | **P-value** |
| Endophytic/Endophytic | >50% exophytic | 12 (27.3%) | 18 (56.2%) | 0.017 | 9 (20.5%) | 19 (59.4%) | 0.002 |
|  | <50% exophytic | 16 (36.4%) | 10 (31.2%) |  | 16 (36.4%) | 7 (21.9%) |  |
|  | Completely endophytic | 16 (36.4%) | 4 (12.5%) |  | 19 (43.2%) | 6 (18.8%) |  |
| Number of septations | Absent | 26 (59.1%) | 9 (28.1%) | 0.006 | 22 (50%) | 9 (28.1%) | 0.001 |
|  | Few (1-3) | 12 (27.3%) | 9 (28.1%) |  | 20 (45.5%) | 11 (34.4%) |  |
|  | Many (>4) | 6 (13.6%) | 14 (43.8%) |  | 2 (4.5%) | 12 (37.5%) |  |
| Septa’s caliber | no septation | 26 (59.1%) | 9 (28.1%) | 0.001 | 22 (50%) | 9 (28.1%) | 0.048 |
|  | Thin (<2 mm in thickness) | 11 (25%) | 11 (34.4%) |  | 16 (36.4%) | 12 (37.5%) |  |
|  | Minimally thickened (3 mm in thickness) | 6 (13.6%) | 2 (6.2%) |  | 5 (11.4%) | 5 (15.6%) |  |
|  | Thickened (>4 mm in thickness) | 1 (2.3%) | 10 (31.2%) |  | 1 (2.3%) | 6 (18.8%) |  |
| Septation nature | No Septation | 26 (59.1%) | 9 (28.1%) | 0.001 | 22 (50%) | 9 (28.1%) | 0.052 |
|  | Regular | 14 (31.8%) | 9 (28.1%) |  | 17 (38.6%) | 13 (40.6%) |  |
|  | Irregular | 4 (9.1%) | 14 (43.8%) |  | 5 (11.4%) | 10 (31.2%) |  |
| Combined  areas of enhancement T1 nephrogenic | No Enhancement | 24 (54.5%) | 3 (9.4%) | <0.001 | 19 (43.2%) | 2 (6.2%) | <0.001 |
|  | One part enhancement | 10 (22.7%) | 6 (18.8%) |  | 21 (47.7%) | 8 (25%) |  |
|  | two parts enhancement | 8 (18.2%) | 9 (28.1%) |  | 3 (6.8%) | 10 (31.2%) |  |
|  | three parts enhancement | 2 (4.5%) | 14 (43.8%) |  | 1 (2.3%) | 12 (37.5%) |  |
| ADC maps based on b500 or 800 | No ADC map | 1 (2.3%) | 7 (21.9%) | - | 3 (6.8%) | 7 (21.9%) | - |
|  | No solid component | 22 (50%) | 1 (3.1%) | <0.001 | 19 (43.2%) | 2 (6.2%) | 0.001 |
|  | Less than renal | 13 (29.5%) | 8 (25%) |  | 9 (20.5%) | 4 (12.5%) |  |
|  | Same as renal | 5 (11.4%) | 5 (15.6%) |  | 9 (20.5%) | 9 (28.1%) |  |
|  | More than renal | 3 (6.8%) | 11 (34.4%) |  | 4 (9.1%) | 10 (31.2%) |  |
| Nodule Presence | | 12 (27.3%) | 26 (83.9%) | <0.001 | 3 (6.8%) | 20 (62.5%) | <0.001 |
| Nodule enhancement on T1 Nephrogenic phase | | 11 (25%) | 26 (81.2%) | <0.001 | 2 (4.5%) | 20 (62.5%) | <0.001 |
| Wall enhancement on T1 Nephrogenic phase | | 7 (15.9%) | 20 (62.5%) | <0.001 | 10 (22.7%) | 21 (65.6%) | <0.001 |
| Septation enhancement on T1 Nephrogenic phase | | 14 (31.8%) | 20 (62.5%) | 0.015 | 18 (40.9%) | 23 (71.9%) | 0.014 |

Table S2. Raw counts and percentages of malignant and benign lesions for each predictive radiologic feature, stratified by reader
